# The Effect of Imatinib Mesylate for Newly Diagnosed Philadelphia Chromosome-Positive, Chronic-Phase Myeloid Leukemia in Sub-Saharan African Patients: The Experience of Côte d'Ivoire

**DOI:** 10.1155/2010/268921

**Published:** 2010-08-25

**Authors:** K. G. Koffi, D. C. Nanho, E. N'dathz, P. Kouehion, R. Dissieka, A. Attia, K. Mozard, A. Tolo, K. Boidy, N. Meité, R. Ayemou, M. Sekongo, N. Tea, I. Sanogo

**Affiliations:** Haematology National Teaching Hospital of Abidjan, 21 BP 632, Abidjan 21, Cote d'Ivoire

## Abstract

Imatinib mesylate, showed encouraging activity in chronic myelogenous leukemia. However, there are few data regarding his efficacy and response monitoring in Sub-Saharan African patients. Our objective was to assess response to imatinib mesylate (Glivec) in Côte d'Ivoire patients with newly diagnosed Chronic Myeloid Leukemia (CML). From May 2005 to September 2009, we treated 42 patients (40 years; range 16–69) with Philadelphia chromosome (Ph+) positive in chronic phase CML with oral imatinib mesylate at daily doses of 400 mg. Overall survival (OS) and frequency of complete or major cytogenetic remission (CCR/MCR) were evaluated. At a median follow up of 32 (range 7.6–113) months, the CHR rate in our study group was 76%. A major CR was found in 19 patients (45%) with 17% and 29% complete and partial CR respectively. There were no significant differences in the incidence of major cytogenetic response by known prognostics factors. Median time to CHR was 8 months (range 0.4–25), and 16 months (range: 0.1–36) for CR. Projected 5-year OS rate was 72% (95%CI 42–88). We conclude that imatinib therapy sub-Saharan African CML patients is very promising and has favorably changed the prognosis for black African patients with CML.

## 1. Introduction

Chronic myeloid leukemia (CML) is a clonal myeloproliferative disorder of a pluripotent stem cell1 first described by John Hughes Bennett in 1845. It was the first malignancy that had a specific chromosomal abnormality uniquely linked to it after the discovery of a minute chromosome now known as the Philadelphia (Ph) chromosome, later defined to result from a t(9;22) reciprocal chromosomal translocation [1]. Of critical importance was the demonstration that this translocation involved the *ABL1 *(Abelson) proto-oncogene in chromosome 9 and the *BCR *(breakpoint cluster region) gene in chromosome 22 [2]. Clinically, CML progresses through three distinct phases: a chronic phase that is easily controlled by conventional chemotherapy followed by an ill-defined unstable accelerated phase, leading to a terminal blastic phase. The latter phase resembles acute leukemia and is highly refractory to chemotherapy with under 20% response rate and a median survival of 3–6 months [3]. Since then, a constant stream of clinical and basic advances has made CML one of the most extensively studied human malignancies. Imatinib mesylate (STI571, Gleevec; Novartis Pharmaceuticals, East Hanover, NJ) is a selective Bcr-Abl protein tyrosine kinase inhibitor against c-abl, bcr/abl, c-kit, and platelet-derived growth factor receptor (PDGF-R). Imatinib mesylate has demonstrated significant activity in all phases of Philadelphia chromosome- (Ph-) positive chronic myeloid leukemia (CML) [4]. Within the past 5 years, a promising new therapy has become available for the treatment of African patients with CML with the first introduction of Bcr-Abl tyrosine kinase inhibitor imatinib (formerly STI571) into clinical trials. With the availability of free imatinib mesylate in resource-poor countries through donation from the Glivec International Patient-Assistance program (GIPAP); this drug has also become the first-line therapy for CML in Cote d'Ivoire a great relief for patients in resource-limited countries. This paper describes the results for patients with newly diagnosed CML in chronic phase.

## 2. Materials and Methods

*Patients.* The national division of Haematology teaching Hospital of Yopougon-Abidjan (CHUY) is the referral centre for diagnosis and treatment of haematology disorders in Côte d'Ivoire. We performed a prospective trial of imatinib mesylate for patients with newly diagnosed African chronic myelogenous leukemia (CML). All patients treated for CML at CHUY have been registered prospectively in our leukemic database, established in 2005. In all patients, the diagnosis of CML was confirmed prior to imatinib mesylate treatment by morphologic review of peripheral blood (PB) and bone marrow and by documentation of the presence of the *BCR-ABL *translocation by conventional metaphase cytogenetic analysis or molecular studies. Chronic phase was defined as fulfilling all the following criteria: (a) peripheral or marrow blasts less than 15%, (b) peripheral or marrow blasts + promyelocytes less than 30%, (c) peripheral or marrow basophils less than 20%, and (d) platelets equal to or greater than 100·10^9^/L [5]. The occurrence of additional karyotypic abnormalities was not considered a criterion for acceleration. The Sokal prognostic score at diagnosis was calculated as described elsewhere [6]. All of the patients gave written informed consent to participate in the studies. 

*Treatment with Imatinib*. In general all patients received imatinib at a oral dose of 400 mg daily; no concomitant chemotherapy was administered other than short courses of hydroxyurea when deemed necessary. Imatinib dosage was adjusted depending on tolerance and response. Imatinib box was delivered to CML patients at the hospital under medical supervision in a full compliance according to GIPAP programs requirements monthly by returning the empty plaques before the next subsidy. Doses were reduced in the presence of grade 3-4 thrombocytopenia or grade 3-4 neutropenia. Wherever possible the dose was maintained above 300 mg/day. The study was approved by the institutional ethics committee and conducted according to the Declaration of Helsinki. Patients with accelerate and blastic phase were not included in our studies. 

Follow-up patients were treated as long as they continued to respond. They were assessed for response to treatment by weekly physical examination, full blood count, and biochemistry for the first 6-weeks and at 6 week intervals thereafter; bone marrow morphology and cytogenetic were assessed every 3 months. Cytogenetic analysis, using the G-banding technique, was performed all patients before the initiation of therapy and every 3 months during the course of therapy. Cytogenetic response was determined based on the percentage of Ph-positive metaphases.

### 2.1. Response Criteria

Cytogenetic responses were classified in accordance with standard UK Medical Research Council practice as complete (0% Ph+ metaphases), partial (1–35% Ph+ metaphases), A major cytogenetic response included complete and partial cytogenetic responses (Ph-positive less than 35%), minor (36–95% Ph+) and no response (96–100% Ph+). 

End points sustained hematologic and cytogenetic response. Hematologic response was defined as described previously. Primary hematologic resistance was considered if patients failed to achieve any hematologic response.

### 2.2. Adverse Events

Adverse events were graded using the National Cancer Institute/National Institutes of Health Common Toxicity Criteria, Version 2.0. Grade 3/4 hematologic toxicity was assessed regarding the time of first occurrence and duration.

### 2.3. Statistical Analysis

Survival analysis was performed using the Kaplan-Meier method. Duration of hematologic or MCR was assessed for responding patients as the time from the first observation of response to disease recurrence, death, or last visit. Overall survival was calculated from treatment initiation to death from any cause or at the date of the last visit. Event Free Survival (EFS) was calculated from diagnosis to the first observation of disease progression or death or interrupted treatment for toxicities. For comparison of cohorts, the Mann-Whitney test was employed.

## 3. Results

Between May 2005 and September 2009, 42 patients with CML were treated with imatinib mesylate (Gleevec). 

The clinical characteristics of all patients are shown in Table 1. The median age of patients was 40 years (range 16–69 years), and 26 patients (62%) were males. The median time from diagnosis was 1.5 years (0.1–7). Risk group (Sokal) stratification for patients with evaluable data was as follows: low risk 16.6%, intermediate risk 44.5%, and high risk 38.9%. Before treatment there were 17 patients (41%) with additional chromosomal abnormalities (Table 2). The overall median followup time was 32 months (range: 7.6–113 months). 

### 3.1. Response, Duration of Response, and Survival

The median time from diagnosis to start Imatinib was relatively long, 9.41 months (range: 0.2–25 months). Overall rates of hematological and cytogenetic responses to imatinib mesylate therapy are shown in Table 3. The median follow-up of the study group was 32 months (range: 7.6–113 months).

**Figure 1:**
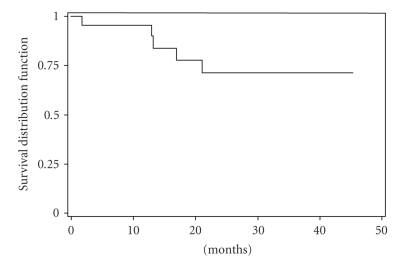
Overall survival of patients with CML imatinib mesylate therapy.

**Figure 2:**
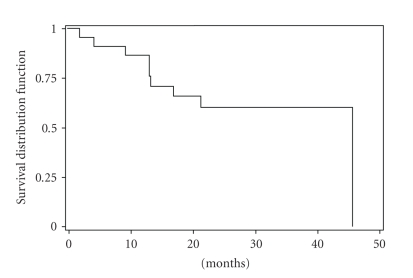
Even-free survival of patients with CML imatinib mesylate therapy.

 The CHR rate in our study group was 76%. A major cytogenetic response was noted in 19 patients (45%): complete in 7 (17%) and partial in 12 (29%). Complete cytogenetic response was achieved in only 1 patient (14%) 3 months of therapy, in 2 patients (29%) 6 months, and 4 patients (57%) 9 months. There were no significant differences in the incidence of major cytogenetic response by known prognostic factors (age, splenomegaly, sex, leukocytosis, thrombocytosis, blasts, and Sokal score). However, during clinical follow-up, patients with additional abnormalities showed significant lower CHR (59% versus 88%) (*P* = .035) and MCyR (24% versus 60%) (*P* = .043) as compared to those with only Ph chromosome (Table 4). But there was no significant correlation between CCyR in the two situations. Moreover, there was no significant correlation between cytogenetic and hematologic response and the delay of treatment. The median time to CHR was 8 months (range: 0.4–25 month), and the median time to cytogenetic response was 16 months (range: 0.1–36 month). The projected 5-year overall survival rate was 72% (95%CI: 42–88) (Figure 1). The projected 2-years EFS was 60 % (95%CI: 35–77%) (Figure 2). This projected 5-years overall survival rate by cytogenetic profile was 78% (95%CI: 38–94%) with Ph+ alone versus 62% (95%CI; 14–89%) with Ph+ with additional chromosomal abnormalities. Side effects of imatinib mesylate in this study were similar to those reported previously for the entire imatinib mesylate treatment study. The severe (grade 3-4) toxic effects experienced are shown in Table 5. At a median follow-up duration of at least 32 months (range: 7.6–113 months) Twenty-three (55%) were alive in chronic phase and are still taking imatinib mesylate, and 10 patients (24%) died. Eight died after developing blastic phase disease; 2 died from pulmonary tuberculosis associated with accelerated-phase disease. Five patients stopped therapy 3 of cutaneous toxicity, 1 with neurological depression, and 1 with cardiac symptoms. No patients died in chronic phase from imatinib mesylate-related complication. Four patients developed accelerated phase and received dose escalated 600 mg daily of imatinib mesylate.

## 4. Discussion

Our study of patients with newly diagnosed CML chronic-phase Ph+ is unique in sub-Saharan Africa. To our knowledge there are few data regarding imatinib therapy for sub-Saharian African patients with Ph + leukemia [7]. Regarding the clinical and biological feature, CML of black African patients seems to be generally aggressive. First, the median age of patients with CML in Côte d'Ivoire and other African countries with similar demographic pattern is over 40 years [7–9]. Fleming et al. reported that native African patients less than 40 years suffer from CML more than any other group in the world; the differential age incidence pattern of CML is believed to be due to the age distribution of African population rather than any other inherent biological characteristics [10]. But this age seems to be lower than that reported in Europe and United states [11]. Second, we found, high incidence of additional cytogenetic abnormalities. So over 41% of additional chromosomal abnormalities were found with high incidence of intermediate and high risk of Sokal staging system length in part with the long mean delay from diagnosis 17 months. The high incidence rate of additional chromosomal abnormalities seems particular in African CML leading to the poor prognostic of our patients. In those studies the percentage of additional chromosome abnormalities length was 15% to 25% for patients in chronic phase [12–14]. 

 With the overall median follow-up time of 32 months (range: 7.6–113 months), we found 76% of CHR rate and 79% had cytogenetic response, which was major in 45% and complete in only 17%. Thus, the results with imatinib mesylate therapy continue to be positive with longer follow-up. This result is lower than that of the reported case of literature. The high rate of RCC was reported by Kantarjian et al. and the IRIS study 81% and 86%,respectively [15, 16]. The study of Muheez et al. in Nigeria reported 35% of RCC **[7].** But in these several studies patients had previously been treated by INF-*α* or other chemotherapy as the first-line treatment. Overall, 80% of newly diagnosed patients with CML in chronic phase would be expected to achieve CCR with imatinib [17]. 

 The median time from diagnosis to commencement of Imatinib was relatively long, t 9.41 months (range: 0.2–25 months), and it is known that this can worsen the prognosis and reduce the probability of response. Our results suggest that there is a small subset of patients whose leukemic cells remain sensitive to imatinib therapy despite the presence of additional chromosome abnormalities. On the other hand, we observed a larger group of patients (12 of 42 patients, 29%) who did not attain a CR with STI571 therapy. This result seems to be high compared to those reported in the literature computer [13, 14]. The karyotype profile of these cases tended to be more complex than the CR cases. Follow-up study on the Ph no-responder CML patients revealed that the majority of patients also failed to respond clinically to STI571 or progressed after an initial brief response to therapy. This can be explained by the higher rate of additional cytogenetic chromosome abnormalities. It appears to be a subset of patients with disease that is refractory to a dose of 400 mg/day of imatinib and who may benefit from dose escalation in the first treatmen intent. The results of this case can be improved by frontline therapy with imatinib mesylate in combination with other drugs or by high-dose imatinib mesylate therapy as previously reported by Kantarjian et al. [17]. 

There were no significant differences in the incidence of major cytogenetic response, by known prognostic factors (age, splenomegaly, sex, leukocytosis, thrombocytosis, blasts,and Sokal systems scoring) perhaps due to the short effectiveness of our study population. In the large randomized study, found the significant correlation between these classical prognostics parameters with the cytogenetic response rate has been [12, 14]. 

Previous experience has led to estimated yearly mortality rates of 10%–15% for patients in chronic-phase CML that had failed to respond or had relapsed during therapy [18, 19]. In our study our mortality rates were estimated as 24%. This mortality rate seems to be very high. 

Imatinib mesylate therapy is now the new standard of care for patients with chronic-phase CML. Combinations of imatinib mesylate with IFN*α*, cytarabine, homoharringtonine, decitabine, or other compounds hopefully will further improve the complete cytogenetic and molecular response rates and thus the long-term prognosis for patients with additional chromosome abnormalities [20]. 

Nonetheless, the projected 5-years overall survival rate of 72% (95%CI, 42–88) seems to be impressive for an African population of patients. The extended IRIS study has recently reported a 5-year overall survival estimated as 89% [16] and several studies have demonstrated significant survival differences based on the Sokal and/or Hasford risk groups at diagnosis [21–24]. This overall survival in this study was better than that of the reported case of our previous study of alpha-interferon with the projected 5-year overall survival rate of 50% [25]. 

 Based on this advantage survival, this work has shown that the outcome of imatinib therapy for newly diagnose Ph+ CML chronic phase in the sub-Saharan African country of Côte d'Ivoire is no far different from reports in the Western populations. We conclude that imatinib therapy sub-Saharan African CML patients is very promising despite the high incidence of additional chromosome abnormalities, with the additional advantage of oral availability and tolerability. Imatinib has favorably changed the prognosis for black African patients with CML.

## Figures and Tables

**Table 1:** Pretreatment characteristics of the study population of 42 patients with CML.

Variable	no.
Gender, *n*. (%)	
Male	26
Female	16
Age (yrs) (median range)	40 (16–69)
Time from diagnosis (yr) (median range)	1.5 (0.1–7)
ECOG at diagnosis	
0-1	25 (59)
2-3	17 (41)
Hepatomegaly *n *(%)	15 (36)
Splenomegaly, *n *(%)	
0 cm	4 (10)
1–9 cm bcma	25 (59)
≥10 cm bcm	13 (31)
Peripheral blood (median range)	
Hemoglobin level (g/dL)	10.2 (5.7–13.7)
Platelet count (10^9^/L)	331.5 (132–1992)
Leukocyte count (10^9^/L)	211.7 (50–854)
Basophils in PB (%)	0 (0–6)
Blasts in PB (%)	1.9 (1–8)
Blasts in BM (%)	1 (0–4)
Ph status before therapy, *n *(%)	
Ph1+	25 (59)
Additional chromosomal abnormalities	17 (41)
Sokal score	
Low risk. *n*. (%)	11 (16.6)
Intermediate risk *n*. (%)	16 (44.5)
High risk *n*. (%)	15 (38.9)

Cma: centimeters below the costal margin.

**Table 2:** Detailed cytogenetic response of 17 CML patients with additional chromosomal abnormalities prior to imatinib therapy.

*Patient n*o.	*Cytogenetic analysis *
(1)	46,XX, t(9;22)(q34;q11)[11] / 45-46,idem, dup (14)(q22;q32)[5]
(2)	46, XY, t(9; 22) (q34; q11) [20]/46, idem, del (12) (p12) [12]
(3)	46,XX, t(9;22)(q34;q11)[9]/47,XX, idem,+8[11]
(4)	46,XY, t(3;13)(p25;q14),t(9;22)(q34;q11)
	[28]/52,idem,+6,+7,+8,+19,+21,+der(22),t(9;22)(q34;q11)[12]
(5)	46, XY, del(3)(p12p14), t(9;22)(q34;q11)[5]/46,XY, t(9 ;22)′ q34;q11)[31]
(6)	46, XY, t(9;10;22)(q34;p14;q11)[15]/ 46,XY, t(9;10;22) (q34;p14;q11),del(13)(q21)[2]/
	47, XY, t(9;10;22)(q34;p14;q11),+mar1[3]
(7)	46,XX, t(9;22)(q34;q12)[29] / 46,idem, t(4;11)(q13;p12)[11]
(8)	46,XY,t(9;22)(q34;q11)[14]/47,idem,+8[11]
(9)	46,XX,t(9;22)(q34;q11),add(13)(p11),add(20)(p13)[23]
(10)	46, XY, t(9;22) ([16]/46,idem,der(12)t(12;14)(q10;q10),add(13)(p11.2),-14,- mar[4]
(11)	46, XY, t(9;22)[12]/46, XY, der(9)t(9;22),der(22q)[7]/ 47, XY, t(9;22), der(22)t(9;22)[11]
(12)	46, XY, t(9;22),-17,add(19)(q13.4),- mar[19]/46,XY, t(9;22),- 17,add(19)(q13.4)[11]
(14)	47 XY,+8,t(9;22)(q34;q11)[3] /46,XY,t(9;22)(q34;q11)[23]
(15)	46, XY,t(9;22)[8]/46,idem,add(1)(p36.3)[12]
(16)	46, XY, add(2)(p25),t(9;22)(q34;q11)[6] /46,XY,t(9;22)(q34;q11)[43]
(17)	46,XY,t(2;9;22)(q15;q34;d11)[28]

**Table 3:** Hematologic and cytogenetic response.

Characteristics	*n*. (%) (*n* = 42)
Hematologic remission	
Complete	32 (76)
Partial	10 (24)
Cytogenetic response	33 (79)
Major (Ph ≤ 35%)	19 (45)
Complete (Ph+ = 0%)	7 (17)
Partial (Ph+ = 1%–35%)	12 (28)
Minor (Ph+ 36% –95%)	14 (33)
Minimal/no (Ph+ >95%)	12 (29)

**Table 4:** Hematologic and cytogenetic response according to Ph+ alone or additional chromosomal abnormalities.

*Characteristics*	*Ph+ alone*	*Ph+ with additional abnormalities*	*P*
	*n*. (%) (*n* = 25)	*n*. (%) (*n* = 17)	
CHR	22 (88)	10 (59)	.0357
MCyR	15 (60)	04 (24)	.043
CCyR	6 (24)	1 (6)	.210

CHR: complete hematologic response; MCyR: major cytogenetic response; CCY: complete cytogenetic response.

**Table 5:** Incidence of grade 3-4 side effects with imatinib mesylate therapy (*n = 42*).

Toxicity	No. (%) with grades 3-4 toxicity
Non Hematologic toxicities	
Bone or Joint aches	11 (26)
Others^(a)^	10 (24)
Skins rash	3 (7)
Nausea	2 (5)
Diarrhea	2 (5)
Muscle cramps	2 (5)
Hematologic toxicities	
Granulocytopenia < 1·10^9^/liter	14 (33)
Thrombocytopenia <50·10^9^/liter	10 (24)
Anemia < 8 g/d liter	5 (12)

^(a)^2 with fever, 1 with neurological depression symptoms, and 1 with cardiac symptoms, fatigue 6.
